# The Prevention of Occupational Heat Stress in Sugarcane Workers in Nicaragua—An Interpretative Phenomenological Analysis

**DOI:** 10.3389/fpubh.2021.713711

**Published:** 2021-10-12

**Authors:** Felipe Pacheco-Zenteno, Jason Glaser, Kristina Jakobsson, Ilana Weiss, Esteban Arias-Monge, Kristina Gyllensten

**Affiliations:** ^1^School of Public Health and Community Medicine, Institute of Medicine, University of Gothenburg, Gothenburg, Sweden; ^2^La Isla Network, Washington, DC, United States; ^3^Faculty of Epidemiology and Population Health, London School of Hygiene and Tropical Medicine, London, United Kingdom; ^4^Department of Occupational and Environmental Medicine, Sahlgrenska University Hospital, Gothenburg, Sweden; ^5^Departamento de Gestión Ambiental y Seguridad Laboral, Instituto Tecnológico de Costa Rica, Cartago, Costa Rica

**Keywords:** occupational health, implementation quality, heat stress, chronic kidney disease of non-traditional origin, climate change, individual readiness for organizational change, interpretative phenomenological analysis, organizational safety climate

## Abstract

**Background:** Chronic kidney disease of non-traditional origin (CKDnt) is an ongoing epidemic that has taken the lives of tens of thousands of people in Mesoamerica, also affecting other tropical geographies. Occupational heat stress, which will increase worldwide as climate change persists, has been identified as a primary trigger of kidney injury and reduced renal function. At Nicaragua's largest sugarcane mill, the water, rest, and shade (WRS) intervention has proven to reduce the risk of heat stress and kidney injury effectively as assessed by the research and policy NGO La Isla Network (LIN) and their academic partners, who have worked with the sugar mill to improve the design of their intervention system. However, discrepancies between intervention design and implementation have been found. This study explores the perceptions of the WRS intervention in the company from the perspective of positions responsible for the workers' environment and heat stress prevention implementation.

**Methods:** A qualitative design was used in the study. Twenty-one key informants of low and middle management, field assistants, and two members from LIN took part in the study. Semi-structured interviews were used to collect the data. Interviews' transcriptions were analyzed using interpretative phenomenological analysis (IPA).

**Results:** Four main themes were developed in the analysis of the data: “A worthwhile struggle,” “Culture of care”, “Traditional production culture Vs. Culture of care,” and “The importance of the formalization of care.” Each theme contained sub-themes, all of which were further discussed in the light of organizational psychology.

**Conclusion and Implications:** Discretionary differences resulting in low and middle management prioritizing production over health protection appeared to relate to a fair part of the implementation challenges and indicate that more efforts are needed to align operations' production and health goals. Education enhancement might be necessary, while further focus on health metrics for performance assessment might offer an opportunity to level perceived incentives and value of health and production.

## Introduction

Rising temperatures due to climate change are making occupational health and safety risks more severe for a large share of the world's working population (1, 2). Heat stress, understood as the combined external heat load and the internal heat generated through muscular work (3), is thus becoming more common (4). In the occupational context, heat stress is more prevalent in countries with *decent work deficits* and often accompanied by other challenges linked to poverty, lack of social protection, and high informality rates (4), which is compounded by inadequate and often misguided development institution investment that contributes to, instead of ameliorates, such labor conditions (5).

Chronic kidney disease of non-traditional origin (CKDnt) is an ongoing epidemic in Central America (6), especially prevalent in the Pacific Ocean coast of the Mesoamerican region (7). The CKDnt epidemic has impacted agricultural laborers dramatically, sugarcane workers in particular, with a death toll that runs into the tens of thousands of people (6, 7). These workers are mostly young men who are affected together with entire communities, as they are usually households' sole source of income in socioeconomically disadvantaged rural settings (6, 8). Recent evidence points that heat-induced kidney injury driven by diverse systemic and kidney inflammation triggers would lead to kidney injury (9), being occupational heat stress from heavy physical work in heat determinant among these triggers in the affected population (7, 9). Thus, implementing occupational heat stress preventive measures is imperative, even more in the face of rising global temperatures (7).

Research on Latino agricultural workers' perceptions of heat-related illness prevention in the US has provided valuable inputs on factors affecting implementation strategies in the workplace. In a study from California, pay structures, employer relations, and subjective worker views (i.e., workers' perceived control, sense of fortitude, and misconceptions about body function) were found to influence workers' self-care beliefs and behavior (10). It was concluded that heat-related illness prevention in the occupational context cannot be viewed exclusively as a biomedical or behavioral issue and, consequently, preventive interventions also need to consider power and control structures existing in the agricultural industry to bring about sustained improvements in workers' heat protection (10). In another study Morera et al. (11) found that workers' perceptions and attitudes of trust toward management contributed to a harvesting operation's safety climate. This underscored the significance of the quality of employer-employee relations and exchanges over other factors such as knowledge of heat-illness prevention. The authors emphasized the difficulties of implementing measures that are not rewarded by the workplace culture. They suggested that, in addition to promoting individual safety behaviors, heat-related illness intervention strategies should promote organizational accountability to increase effectiveness (11).

Furthermore, the piecework payment structure has been associated with an increased risk of agricultural injury compared to other payment forms (12–14). Among Latino agricultural workers in the US, piecework has been identified to affect the adoption of heat safety practices, as manual workers “.have a financial incentive to boost their productivity with faster, less careful work and to avoid interrupting it with water and restroom breaks.” [(11): p. 2]. Moreover, in the study from California, foremen were perceived differently by manual workers depending on their pay structure (10). Supervisors' directives on heat safety practice were perceived as impositions when paid piecework and “acts of compliance” when paid hourly [(10): p. 18].

On the other hand, many change efforts fail in organizations as individuals' central role is often underestimated by change leaders (15). The individual readiness for organizational change (IRFOCH) has been identified as critical to the successful change implementation in organizations (16, 17). Holt et al. (16) defined it as “the extent to which an individual or individuals are cognitively and emotionally inclined to accept, embrace, and adopt a particular plan to purposefully alter the status quo.” within an organization [(16): p. 235]. Individual readiness for organizational change considers individuals' beliefs of: (a) their change-specific efficacy, as individuals' believe that they are capable of implementing a proposed change and feel confident that they would perform it successfully; (b) appropriateness, which is the belief that the proposed change is appropriate and would be beneficial for the organization; (c) management support, as individuals believe that the organization leaders support the change; and, (d) personal valence, as they believe that the proposed change is beneficial to the organization's members (16). Furthermore, individuals' evaluation of how organizational infrastructure can facilitate organizational change efforts will affect their readiness for the change (16). The initial readiness for change (18) encompasses, in turn, readiness both at the individual (IRFOCH) and organizational levels. At the macro or organizational level, structural factors will define the circumstances in which change occurs and the degree by which those circumstances will enable or hinder change implementation (19). Then, individual and structural factors of initial readiness would simultaneously influence change implementation at the individual and organizational levels (20).

While a company's management system can be considered its formal part, the organizational culture can be seen as its informal component (21). The culture in an organization refers to the values, beliefs, and attitudes that the different individuals or members of the organization share, which can emerge both at the higher, or organizational level and among smaller groups within the organization (22). The organizational culture comprises the basic underlying or deep-level assumptions and values that become manifest in practically all aspects of the organizational life (21, 23). On the other hand, the organizational climate construct describes, at a more superficial level, the perceptions that employees share with respect to the procedures, practices, and behaviors that get rewarded and supported in relation to a specific strategic goal (24). When this strategic goal involves practices or behaviors that entail certain health risk for employees in a company's operations, the shared perceptions about this define organizational safety climate (25, 26). Employees perceive signals regarding policies from both top management and direct supervisors, regarding how those policies will be operationalized in their immediate job contexts (27). When discrepancies emerge, employees perceive a lack of internal consistency between the organization's policies, procedures, and practices at their particular departments or units (25).

It has been suggested that companies that manage the health and safety of their employees adequately obtain better economic outcomes from their operations than if they managed solely on production metrics (28). However, many business leaders have an implicit and unfunded belief that injury risk prevention at the workplace requires a trade-off between profits and the expenses associated to health and safety (28). Zohar (25) described that, in practice, regulations and procedures associated with safety policies at the workplace tend to compete with other domains such as productivity or efficiency. In this situation, employees are confronted with the challenge of discerning what behaviors are expected, rewarded, and supported out of “the overall pattern and signals sent by a complex web of rules and policies across competing domains” [(25): p. 1518]. The distinction between identification of priorities and espoused and enacted priorities is of significance for the understanding of safety behavior because only managers' espoused priorities inform employees of “behavior-outcome expectancies” (25). Bowers et al. (29) described employees' perception of top management commitment to safety in terms of (1) top management's active participation in safety; (2) allocation of resources to safety; (3) inclusion of safety in the structure of the organization, and (4) consistency in actions and decision-making.

### The Water, Rest, and Shade Intervention at Ingenio San Antonio

At Nicaragua's largest sugarcane mill, Ingenio San Antonio (ISA), the Adelante Initiative, a multi-stakeholder platform formed by the NGO La Isla Network (LIN), the sustainability certification group Bonsucro, Nicaragua Sugar Estates Limited, The Nicaraguan Sugar Producers Association, the German Investment Corporation, and ISA, aims to assess the impact of evidence-based recommendations, designed to improve working conditions and reduce kidney dysfunction among sugarcane workers (30). Built-up upon existing efforts to prevent heat stress at ISA, in 2017, the water, rest, and shade (WRS) intervention was rolled out under the Adelante Initiative framework. In this intervention, workers are required to take regular breaks under movable tents installed to provide shade, and have access to liquid, particularly water and a specially developed electrolyte solution (31). The Adelante Initiative has focused on burned sugarcane and seed cutters. These two jobs have the highest physical demands among various other manual jobs at the mill and have been considered a priority for risk mitigation and assessment (31). The WRS intervention has been proven to effectively mitigate the risk of heat stress and maintain sugarcane workers' renal function (7, 30).

Moreover, the WRS intervention entails benefits for both workers and employers, which is the foundation for a sustainable production model (30, 32). Under a conservative estimate of the average net gain in productivity, a 22% return on ISA's investment in the WRS intervention was obtained (33). Besides, the German Investment Corporation has been granting simultaneous loans to ISA for their production operations and the enforcement of their occupational health and safety structure through the Adelante Initiative. This combination has implied a strong incentive for the mill to continue with the implementation of heat stress prevention.

However, despite considerable progress toward ensuring workers' heat safety with the WRS intervention, there were discrepancies between intervention design and the quality of the implementation at ISA (30). Clearly, effective implementation processes are an essential step to ensure heat stress prevention, and there is a lack of knowledge regarding how psychological and organizational factors affect the implementation of the WRS intervention.

Therefore, this study aimed to fill this gap by exploring the perspectives of individuals that, in different ways, are responsible for the workers' environment and the implementation of the WRS measures. Additionally, the study's findings could. be relevant for other interventions addressing occupational heat stress in similar settings within industrial agriculture and other productive sectors.

This qualitative study explored the perceptions of the WRS intervention and its implementation at the ISA sugarcane mill from the perspective of key informants at low and middle management levels and field assistants. The research questions were:

a) What are the main enablers, obstacles, and opportunities for improvement to the implementation of the WRS intervention?
b) How do low and middle-level management and field assistants perceive and experience the WRS intervention, as well as their roles, and other organizational levels' role, in the implementation process?

## Materials and Methods

### Research Design and Location

The study used a qualitative design with an interpretative phenomenological approach (34). This was considered the most appropriate approach to assess individuals' perceptions and experiences of the WRS intervention and how they make sense of it, personally and in relation to others at the workplace. The study was conducted in collaboration with LIN and ISA's management as part of the Adelante Initiative's sub-studies, aiming to evaluate the quality of the WRS implementation. Individual semi-structured in-depth interviews were used to collect the data, as this is the method that is well-suited with an interpretative phenomenological approach (35). The interviews were conducted in February 2020 by one of the authors (FP-Z) in Chichigalpa, western Nicaragua, at the participants' workplace in the sugarcane fields and ISA's facilities.

### Participants, Sampling Procedure, and Sample Size

**Figure 1:**
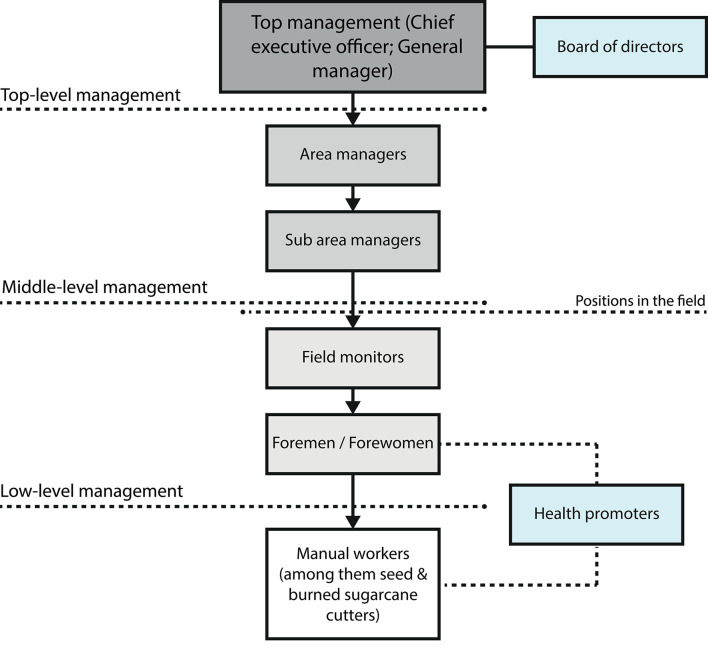
Organizational levels and positions at ISA.

A member of LIN coordinated the selection of participants. From a preliminary list of all the individuals holding positions responsible for the manual workers' environment, and therefore relevant for the study, the selection of participants was done through a convenience sampling in terms of the individuals who were working at the time the researcher (FP-Z) was visiting the fields. The selection process was inspired by maximum purposeful variation sampling to allow a broad spectrum of personal and contextual preconditions to be explored. In order to increase the likelihood of acquiring rich data in terms of various empirical accounts of experiences, the participants selected held positions in different administrative areas of ISA (Agriculture, Operations and Harvesting, Human Resources, and Occupational Health and Safety) and at different organizational levels (see Figure 1). Even though randomized sampling was not feasible for the study, all positions that are directly related to the implementation of the intervention in the field were interviewed.

Low management participants (field monitors and foremen/forewomen) and field assistants (health promoters) work either all or most of the time in the fields, while middle management participants work mostly in the company's offices (area managers and sub area managers).

In total, 23 individuals participated in the study, 16 men and 7 women. This is in line with the recommended sample size of 10 individuals for the method interpretative phenomenological analysis (IPA) (34). Six foremen/forewomen (four men and two women), four area managers (all men), four sub area managers (three men and one woman), four field monitors (all men), and three health promoters (all women) took part in the study. Additionally, two key informants from LIN were selected (one man and one woman). They held positions as a researcher and in-country operations within this organization.

Ingenio San Antonio's manual workers belong to a socioeconomically vulnerable population to which limited opportunities for finding stable employment are available (6, 8, 36). Likewise, this group has particularly low educational levels compared to other population groups in Nicaragua. As of the Adelante Initiative's baseline data collection (36), 18% of the surveyed burned sugar-cane cutters had no formal schooling, and 57% had not completed primary school. These and the other manual workers find a relatively stable employment opportunity at ISA, where they are hired directly by the company, on temporary contracts for 6 months a year (the harvest season's length), and where they often return for several years to work in the fields (36). The compensation system established for most manual workers is a piecework payment structure, which differs from all other positions above them. Their immediate supervisors (foremen and forewomen) are paid hourly or under other agreements on a stable basal salary. Since heat stress prevention implementation started at ISA, functions regarding preventive measures' compliance were added to foremen's and forewomen's responsibilities. Currently, they are responsible for meeting the production targets set for the manual workers they supervise and must cooperate with the health promoters to ensure access and compliance with the WRS prevention measures (37). Health promoters are responsible for executing heat stress prevention for various kinds of manual jobs in the field. They help ensure workers have access to water, move tents, and offer training to manual workers during their breaks on the importance of WRS, and other health-related topics (37). Health promoters are predominantly female, which contrasts with a male predominance in all other positions explored. This indicates that roles are essentially gendered in this setting. Mainly women carry out care and education tasks while men hold most leadership and production positions. Lastly, field monitors perform different supervisory functions, such as guiding on technical matters (e.g., agronomic procedures or quality of cutting) and overseeing the coordination in the field. Further in the text, the word “foremen” will refer both to men and women in this position. Similarly, the focus will be exclusively on the two manual jobs prioritized in the Adelante Initiative, seed and burned sugarcane cutting. The manual workers performing these jobs will often be referred to as “cutters.”

### Sources and Data Collection Procedure

The interviews took place at the participants' workplace in the sugarcane fields and the company's facilities. Only places that allowed privacy were used. Seven individuals with whom it was not possible to speak in the sugarcane fields for practical reasons were interviewed on the last day of data collection in a private office at the mill's facilities. The interview guide was developed on the basis of theories of individual and organizational change and comprised questions that explored: (a) The main perceived enablers, obstacles, and opportunities for improvement to the implementation of the WRS intervention; and (b) How do low and middle-level management and field assistants perceive and experience the WRS intervention, as well as their roles and other organizational levels' role in the implementation process. During the interviews the interviewer asked follow-up questions and the participants were asked to expand on their answers. The interviews lasted between 30 min and 1 h. All interviews were tape-recorded and transcribed verbatim in their original language, Spanish.

### Analysis

The data were analyzed using IPA, following the guidelines of Smith et al. (34). Interpretative phenomenological analysis aims to explore in detail how participants experience and make sense of the topic under investigation. This method is a phenomenological and interpretative approach, where the researcher initially develops an “insider's perspective” on the topic while also offering an interpretative account from an “outsider's perspective” (34).

After transcribing the data, one of the researchers (FP-Z) worked with the text: (1) doing initial coding, (2) identifying shared themes, (3) analyzing shared themes, (4) searching for patterns, connections, and tensions, and (5) writing up the final list of clustered themes and sub-themes, a recursive process in which themes and sub-themes suffered modifications as the process of writing is an essential part of the analysis in accordance with IPA (34). In order to strengthen inter-rater reliability, one of the authors (KG) read the quotes and checked the generation of themes throughout the whole analysis process. Consequently, some of the themes were revised until a final list of themes was agreed on. Steps 1, 2, 3, and 4 were done with the software Atlas.ti (version 8.4).

### Ethical Considerations

The names of the participants have not been used in the analysis. Instead, aleatory numbers have been assigned to each interview transcription. Information that would lead to identifying specific individuals or sensitive comments is not included in the quotes or the analysis. All data (audio recordings, transcripts, and analysis records) are stored and handled according to Swedish regulations.

## Results

Four main themes and a number of sub-themes were found in the analysis, see Table 1.

**Table 1:** Clustered themes and sub-themes.

Theme 1: A Worthwhile Struggle	1.1 Perception of being already used to/adapted to the implementation of the preventive measures 1.2 Different beneficiaries of the WRS intervention 1.3 Pride on being forerunners and the expectation to maintain and improve results 1.4 Potential benefit of communicating and spreading information on the implementation within the company
Theme 2: “Culture of Care”: The Importance of Education and Follow-Up	2.1 To need for the managers to become aware and to raise awareness in others 2.2 The need for the cutters to become aware 2.3 Follow-up in the field and controlling for compliance 2.4 Foremen as key actors
Theme 3: Traditional Production Culture Vs. Culture of Care	3.1 Tension between two ways of producing 3.2 Importance of not affecting the production goals adversely 3.3 Task overload in the field 3.4 The need for a greater involvement of low and middle management
Theme 4: The Importance of the Formalization of Care	4.1 Recording and monitoring health indicators, and measuring impact 4.2 A committed top management 4.3 The weight of guidelines and regulations 4.4 An organizational structure that facilitates decision-making, supervision, and coordination on health protection 4.5 The importance of having key performance indicators based on health outcomes

### Theme 1: A Worthwhile Struggle

#### 1.1 Perception of Being Already Used to/Adapted to the Implementation of the WRS Preventive Measures

Foremen and health promoters described their work as a daily struggle. According to all foremen, their struggle was most difficult at the beginning of the implementation of heat stress preventive measures. Even before the Adelante Initiative, heat stress prevention requirements initially felt like an extra load. A few foremen expressed that they sometimes did not believe that they would overcome this challenge.

“*The truth is that I felt it* was quite *heavy. And that is why maybe, sometimes I got to the point of thinking, ‘we are not going to make it, we are not going to make it.' Because I used to say, ‘it is discouraging to be fighting all the time,' because for each thing, for each assigned task you have to fight with the people* [the workers]. [With] *Fight I mean to do it, to* [make them] *comply with it* [the WRS preventive measures].”

All participants described that not knowing why there were so many sick workers had been very difficult, and the individuals that had had the most contact with the cutters appeared to have been impacted the most. According to the foremen, the emotional burden seemed to be, for the most part, overcome, and they now appeared to be able to make sense of the causes of the situation. At present, the dissimilarity with the past scenario is, to some participants, exciting.

“*I mean, the truth is that this* [the WRS implementation's results] *is something exciting... Because before we used to say, ‘Poor guys!' ‘This is terrible!' ‘So many sick people!'. That is what we used to say. Why? Because they put up with being thirsty. They did not drink the very necessary liquid they had to drink and we* [were] *sad that... ‘So many sick workers!', as we used to say, ‘What is happening?'*”

The hard work on implementing the WRS preventive measures in the field has yielded positive results as the situation is currently considerably better. Many participants from all organizational levels expressed a feeling either of satisfaction or pride over the results they have obtained. All interviewees in the mill said that implementing the WRS intervention feels like part of normality at present.

#### 1.2 Different Beneficiaries of the WRS Intervention

All participants emphasized the benefits to cutters' health and work continuity. Some participants also valued the benefits for workers' families and the communities. Likewise, foremen expressed that they have also benefited from the intervention, as they are now taking more care of their own health in the field.

“*It* [the intervention] *is very good because this way at least you find out how you are doing, because that is important! Sometimes we need it, we who are in the sun, in the field, every day... ...It gives us the opportunity to take the* [medical] *tests, it gives us information on how we are doing. For me it is something very important*.”

To middle management participants, the WRS intervention had brought positive benefits for both the workers and the company, as the latter can now produce at similar levels as previously, but in a sustainable manner.

“*Better conditions generate greater productivity, greater efficiency, and therefore I believe that it is a win-win project. This is how I would summarize it*.”

#### 1.3 Pride on Being Forerunners and the Expectation to Maintain and Improve Results

All participants expressed their wish that the implementation should continue and said to be open to further improvements. To all participants who work in the field, expectations centered on maintaining the benefits seen in cutters' and their own health. At the middle management level, it was also recurrent to emphasize the pride they feel on being forerunners in better work practices in the region and their expectation of strengthening ISA's reputation as a model mill. According to one area manager, the added value for the company in terms of external recognition motivates production areas to get involved with the WRS intervention.

“*Things are changing because the mill has benefited... We benefited a lot because we have the recognition of almost the entire industry and we have been awarded as a model mill in terms of sustainability, in good practices for production. And that also motivates the production engineers. ...people are changing, and they start owning this, and they get more and more involved*.”

### Theme 2: “Culture of Care”: The Importance of Education and Follow-Up

All participants expressed that it has been necessary to achieve a change in the traditional culture, centered solely on the production, toward what many called the knowledge or awareness of the importance of caring for health or the “culture of care” (*cultura del cuido* in Spanish). To positions at all organizational levels, much progress has been made in what foremen and health promoters called the “adaptation to care,” and some managers expressed as the change occurred toward “a new way of producing.”

Continuous education in the field was pointed out as the most significant facilitator to break down the perceived cultural resistance to caring for health and as the primary vehicle for awareness and engagement with the preventive measures. At the same time, permanent supervision and control, with eventual sanctions, have been required. All these elements form what participants called the “follow-up” on the cutters, which for them, is a component of the “culture of care.” All participants identified education and follow-up as fundamental enablers to an effective implementation.

“*There are two lines: one is to make people aware that it is for their good, and the other is the supervision part, which is that, well, when they* [the workers] *are losing the lines, then we align them*.”

#### 2.1 The Need for the Managers to Become Aware and to Raise Awareness in Others

Most participants at all organizational levels expressed that the individuals in charge of those workers who have to become aware must experience this process first to be able to “transfer” the awareness and support the implementation.

“*Supervisory staff must be made aware first so they accept it first, so that they can pass it on to the fieldworker. Because if we, the supervisors, cannot first absorb the benefit that the program has, we will not be able to transfer it*...”

With variations in where the participants' emphasis was placed, three elements appeared to form the process of becoming aware in the person in charge: (1) The awareness or knowledge of the relationship between heat stress and kidney complications. (2) The verification from their own observation that the WRS preventive measures effectively mitigate them; this is, the preventive measures result in health benefits. (3) The verification, also from their own observation, of the maintenance or improvement in the production. According to many participants, this awareness process allowed them to get involved, control, and promote the “culture of care” in their dependents throughout the implementation.

“*One has to take it in* [the intervention], *and then, how shall I say this, express and share one's doubts with the Occupational Health Department, that one has to look after production, because we have to watch the production of the company, right? And while internalizing this test with them* [OHS Unit]*, we realized that it worked. So, I would say that ‘yes, we have to try this...'.But first we had to internalize this test and we had to do it before them* [the cutters], *because otherwise we would not contribute in helping, supporting them*.”

#### 2.2 The Need for the Cutters to Become Aware

Even though considerable progress has been made, according to all participants, making the cutters aware is an ongoing challenge. To most participants, the cutters appeared to be viewed like children who disregard their health and, hence, need to be reminded repetitively to become aware that their health is important.

“*The first effective element is the daily insistence on awareness. ...We say here that the guys are like kids that you have to be telling every day: ‘Hey, put the clothes in the laundry basket!' ‘Hey, brush your teeth!' ‘Hey, put your shoes on!' So, daily! That is the element that will make you successful*.”

The vast majority expressed that the obstacle for this awareness is educational or cultural, an obstacle that translates into not being (at least not wholly) aware of the health risks they are exposed to and not taking appropriate care of themselves.

“*I think that the biggest obstacle that we as people, as cutters, as staff have is the education. With that basis, well, we are not aware of risks. ...Therefore, that* [lack of] *education is what we address with day-to-day training. ...when they* [cutters] *become aware* [of the risks]*, then that obstacle starts to decrease. ...But basically, in my opinion, the first obstacle is that people must become aware of taking care of themselves*.”

#### 2.3 Follow-Up in the Field and Controlling for Compliance

Even though there was no consensus on whether the intervention would be complied with if the WRS preventive measures were not mandatory, all participants highlighted that constant supervision and control in the field are necessary for a high compliance level. An area manager emphasized his/her perception of cultural factors affecting this need to supervise and control compliance. In addition to cultural factors, a few participants from all organizational levels understood this need regarding staff turnover.

“*If we do not supervise, the program fails. We are not as disciplined as the Swedes, we have to take people by the hand and make sure things are being carried out day by day and all the time. Despite the fact that there is an awareness already created, we always need to control that the rules are followed*.”

To ensure compliance with the preventive measures and other safety requirements, foremen can sanction the cutters if they disobey, first, through a written warning in their files, and later with days of no work if they refuse to comply again. Mostly for health promoters, those sanctions appeared to be important for their work and essential for the WRS intervention.

“*I think it is effective, they* [punishments] *are good because they are corrective*. *Because if they... if they, let us say, as a company, as chiefs, would not apply those punishments to the worker, we would still be* [in] *the same* [situation] *as before, honestly*.”

All participants said that compliance with the WRS preventive measures requires a constant follow-up on health. Foremen and their supervisors expressed that knowing that they are permanently monitored helps cutters and themselves take care of their health.

“*People already know that they are visited* [in the field] *frequently, that they are watched and that they have to put in more because they are monitored periodically, continuously. So, you as* [supervisory] *staff, you know that you should also take care of yourself because of the same reason*.”

According to some participants, from all organizational levels, the follow-up on health indicators, the supervision and control, and the constant repetition sooner or later result in the establishment of new habits in the cutters.

“*In the end, it has a psychological impact because the person gets used to it. And we have seen improvement, indeed!”*

#### 2.4 Foremen as Key Actors

All participants agreed that foremen are key actors to ensure compliance with the WRS preventive measures.

“*And yes, as I say, the one we must insist to work strongly on this* [making cutters aware], *is the one who spends the most time with them* [the cutters]*... ...the foremen. Those are the people who have to be more persistent. …...It is them you have to rely on more for compliance*.”

All foremen highlighted the significance of their role and social ability necessary to ensure that cutters, who have different personalities, become aware of the importance of caring for their health and following the WRS preventive measures.

“*And that is what we are for, to influence, right? And to teach them to take into account that this is for their own good*.”

On the other hand, while highlighting the crucial role foremen play, one sub area manager pointed to the need for them to receive even more training regarding the implementation of the WRS preventive measures, given the cultural and job responsibilities' changes they experience.

“*At the beginning it was quite hard because, well, even for them* [foremen] *this is a cultural change. Previously, they only walked around watching the people* [cutters]*, right? Now it is something else, one more detail. …...but yes, they are key, they should be given even more training for that*.”

Accordingly, most foremen expressed their wish and need to get more support in supervising the implementation and in their educational work with the cutters. A few others pointed to the need to receive more training, motivation, and support for themselves.

“...*that someone would come to supervise us. Maybe…... that someone would come to explain, not to tell you, but to explain. How do I say this, that someone encourages you a little more. Give us a bit more talks, I mean, training*.”

Nevertheless, a few participants at the middle management level emphasized the importance that the positions above foremen should also be engaged in health protection so that foremen can perform their decisive roles in the implementation. One participant from LIN expressed that the foremen will not do the logistics in the field needed to ensure full compliance if field monitors and the middle-management are not committed.

“*The logistics will be achieved starting with the foremen. Then the first issue must be their commitment. ...I think they* [the company] *should start with the chiefs, the foremen... They will guarantee it* [the compliance]. [However]*...if their supervisors, -the immediate heads of the different areas-, are not committed, they* [foremen] *will not implement the logistics as defined* [by the protocols].”

### Theme 3: Traditional Production Culture vs. Culture of Care

#### 3.1 A Tension Between Two Ways of Producing

According to one area manager and all LIN's participants, there is still a tension between the traditional focus on production indicators and the incorporation of health protection measures in the production process. The “new way of producing,” as mentioned in theme 2, seemed to be challenged by some organizational members' exclusive focus on productivity.

“*There are still certain barriers in this regard, even though there is an order from above which is what must be followed. There is still a bit of pride among the engineers who do not want to understand that it is necessary to also take care of the health of the workers, and not only to produce*.”

The same area manager expressed that production areas might influence the staff toward the production rather than the implementation, which, to him, serves as an obstacle to the latter. Additionally, one participant from LIN illustrated the difficulty of integrating heath protection for some positions in the mill. This focus on production appeared to be related to a lack of commitment.

“*Often the* [production] *departments see the safety system of the workers as a barrier to their production, so this* [the incorporation of the WRS preventive measures] *is very difficult for them. So, I tell you that the managers of the areas have to be committed, if the manager is not committed, he sees it as a barrier to his* [area's] *production*.”

#### 3.2 Importance of Not Affecting the Production Goals Adversely

All the participants expressed the importance of the recently confirmed fact that the WRS preventive measures do not affect the production adversely. The knowledge or awareness of this was understood as a crucial facilitator to the implementation. Consequently, all participants mentioned that the main obstacle in practice for the cutters to comply with the preventive measures had been their concern that following the measures would decrease their personal production and, therefore, their salaries. Hence, an educational aim has been to inform the cutters that they do not lose with the WRS preventive measures but, instead, win in health and can keep their former production and salaries.

“*It has been a bit slow because we have had to teach, to educate, -due to the* [work] *culture we had, even the* [higher positions'] *staff-, that this is for their benefit. We had to be able to instill the culture of rest and care, of personal health. Due to this cultural issue, there are those who say: ‘No, if I rest, I do not earn* [money]*.' But it has been the opposite, they have been able to earn while also gaining health*.”

Many participants of low and middle management said that, in the beginning, they have also resisted the implementation because of the belief that this would affect the production, while also highlighting that this obstacle no longer exists.

“*I believe that we no longer have fears, those fears have already been overcome and rather, I believe that what is coming and all the changes that might come we are going to assimilate them in a better way; because we have already managed to overcome the cultural obstacles that we had, those of production*.”

Although participants expressed that income concerns do not hinder the great majority of cutters at present, one participant described that a current obstacle for some cutters relates to the piecework pay system, while noting he/she does not experience this difficulty personally, as he/she is paid hourly.

“*So, sometimes people, with these breaks they say: ‘We are going to earn less.' Because that is the first thing they say. I mean, the ones who make their salary by piecework. We don't, because we have a stable salary*.”

#### 3.3 Task Overload in the Field

The vast majority of foremen and health promoters referred to the *boleros* (specialized operators who initially supported the implementation in the field but later were withdrawn) and expressed the need for such support. A foreman expressed to experience difficulties due to the number of tasks corresponding to both production demands and heat stress prevention measures:

“*Well, I don't know*, [it would be better] *that someone else could help us. There is the support we have here but… ...someone to make the work a little lighter, because I am doing two things: I am there supervising the cutting and I am the helper of the* [health] *promoter, and … move the tent, put the water, refill the water. A little more. someone to help*.”

Furthermore, one participant from LIN pointed out that an obstacle to achieving full compliance with the WRS preventive measures is the current heavy workload of foremen. Another LIN participant highlighted that health promoters also experience task overload.

“*The foreman is in charge of monitoring the quality of the work* [the cutting of the cane] *and in addition to that, he has to check the installation of the tents, that the tents are moved, that there is always water is in the thermos… To me, there is work overload, I mean, if you focus on the water, you neglect* [work] *quality, if your boss arrives, then he starts scolding you or asking you why the quality of work is decreasing. ...if you focus on quality, then you drop the part of water, rest, and shade*.”

#### 3.4 The Need for a Greater Involvement of Low and Middle Management

One managerial-level participant described aspects that could be improved in the implementation; among these, the need for greater involvement of the middle management (sub area managers), field monitors, and foremen.

“*All heads of specific areas and their structure of supervisors and foremen, we train them too and involve them in everything we do as Occupational Health, but I think we have to find how this information can reach them in a better way and more continuously. It is necessary to find another method so that they get directly involved*.”

Similarly, the same participant pointed out that there are foremen that are empowered to implement the WRS preventive measures and other foremen who still need to become more empowered.

“*.we also realize that this is because there are good foremen, they are very empowered. So, we need all foremen to be empowered, like the model foremen we have.”*

### Theme 4: The Importance of the Formalization of Care

#### 4.1 Recording and Monitoring Health Indicators, and Measuring Impact

Middle management participants and field monitors pointed out that, although the company had health prevention measures in place in the past, the difference and advantage today lie in the formalization of health indicators' recording and monitoring in the field, as well as in the regularity and organization of the preventive measures according to this monitoring. Furthermore, they emphasized that impact measurements have been essential to consolidate the implementation and continuity of the WRS preventive measures.

“*For decades, the production was done in such a way that the worker was given his water, his protective equipment, but the impact the work or the climate* [weather] *conditions could have on his health was not measured*.”

#### 4.2 A Committed Top Management

At all the organizational levels explored, especially to middle management participants, the top management emerged as highly committed to the WRS intervention and its implementation. To all participants, this commitment is what ensures the setting-up, development, and continuity of the program. A sign of top management's commitment is the follow-up they continuously do. Some participants highlighted, as well, the creation of the Occupational Health Management as proof of top management's engagement in health prevention and support to the program.

“*I would say really support, unconditional support to the program, definitely. Of that there is no doubt. This and the follow-up, the monitoring of the program is not something that has stayed like: ‘Hey, do it', and that is it. No: now it is like: ‘How are we doing? What needs to be improved? What do we need to keep as it is?' Even a management area* [OHS Department] *was created to give more follow-up to this issue. ...There is a lot of interest, a lot of interest*.”

All participants expressed that the directives that come from the top management motivate all that is done at each level of the chain of command until the lowest level. Likewise, the directives originating from the top management, in participants' accounts frequently reflected feelings of support.

“*So, I think that this is the most important, right? To always have the support of the higher positions and that the cutter feels that we, who are the lowest in the upper positions' team, also support them, because that is what we are here for. ...The one at the top has given the instructions, until we reach the “highest little one”* [foreman] *to share them* [the instructions].”

#### 4.3 The Weight of Guidelines and Regulations

All participants emphasized that the company has guidelines and regulations that endorse and support the program. These appeared to be perceived as the base from where everyone involved in the implementation articulates the coordinated effort necessary to achieve success with the WRS intervention.

“*And that is our priority, not only for us as supervisors* [foremen]*, but also Occupational Health, which is also our boss. They are* [coordinating] *like: ‘Okay, alright, it is this, and look at that, okay, okay*.'* We are all interested in complying, complying with the regulations that guide us and getting to the point, well, of being successful, as we are now*.”

Health promoters highlighted, as well, the significance of the regulations, which give them the formal support and authority to perform their control role, as well as facilitate their communication with foremen. To them, regulations' enforcement indeed enables that cutters comply with the preventive measures at present.

“*Before they* [the company] *did not enforce so much the regulation. The worker, well, did not comply much with the regulation....But now with all the rules.”*

Some foremen, however, expressed to have abided by the directives they received from above without having any choice, regardless of the fact they said to follow them willingly.

“*So, they give directives, well, the staff* [upper positions] *give us a directive, so we have to abide by it and make them* [the cutters] *comply with it*.”

“*We have no choice but to take it forward, in any case*.”

#### 4.4 An Organizational Structure That Facilitates Decision-Making, Supervision, and Coordination on Health Protection

According to some middle management participants, moving up the OHS Department to a position equivalent to the Human Resources Department has notably facilitated the implementation. At the same time, an executive committee to follow-up on the company's occupational health prevention measures (where top-management/decision-makers participate) has been settled. This has implied a strategic success toward the implementation, according to one area manager. After this change in the organizational structure, the OHS and HR departments work together and at the same level of authority to design and control the company's implementation of the WRS preventive measures.

Also, according to a few participants of middle management positions, the recent change of field staff (cutters and foremen) from the administrative dependence on production areas to the HR Department has been crucial to facilitate the effective implementation of the preventive measures. As expressed by one area manager, the supervision of the foremen who are under the HR leadership is now smoother as they follow instructions easier.

“*For Cane Cutting it has been easier because those foremen depend* [administratively] *on Human Resources, and it is easier to make them follow instructions. In other areas, they do not depend on us, they depend on the production departments*.”

#### 4.5 The Importance of Having Key Performance Indicators Based on Health Outcomes

A few area managers illustrated the importance and helpfulness of having key performance indicators based on cutters' health results. Currently, performance evaluations in the company include health results to some extent, and only per area.

“*If I go to the field, for example, and I see a worker who does not use some protective equipment, no matter how minimal this is, I will go and tell him: ‘Put it on, if not, go sit there, but don't continue working.' Why? Because it is preferable to pay that man a day without working* [As some field jobs are paid per working day] *than that something would happen to him, because this affects my* [area's] *indicators*.”

According to one area manager, having specific key performance indicators based on cutters' health results would generate a greater commitment to the program among the middle-management, field monitors, and foremen.

“*To the extent we* [mill's management] *evaluate all those foremen, evaluate their performance based on the results of people's health, they will become more aware of the importance this has*.”

“*I think that their immediate bosses* [of foremen] *should ensure that their performance goes beyond the fulfillment of filling a cane sack or cutting a cane furrow, a row. Their* [foremen'] *performance* [assessment] *should also evaluate that no one dehydrates, that no one fatigues. …To ensure that performance at each level of supervision, at all intermediate levels, also includes guaranteeing that no one gets sick*. [Currently] *Contributing to the implementation is a clause in their contracts and it is mentioned in their job profiles, but I think it needs more emphasis. We must find a strategy so that they understand that it is a requirement that they must comply with and something that is subject to your* [their] *responsibilities as an employee in the company*.”

### Discussion

The study aimed to explore individuals' experiences of implementing the WRS intervention at the ISA sugarcane mill. Specifically, among participants of low and middle management and field assistants. Four main themes were developed in the analysis: “A worthwhile fight,” “Culture of care,” “Traditional production culture vs. Culture of care,” and “The importance of the formalization of care.”

In the first main theme, “A worthwhile fight,” it was highlighted that the WRS intervention had been successful in many respects despite the participants describing that they initially had been unprepared for the implementation. Seeing sustained benefits from their work on the implementation and being part of the efforts to decrease the number of sick workers were described as motivating to continue with the interventions. This might have contributed to beliefs of appropriateness, and personal valence (i.e., the change is beneficial for the organization's members) (16) toward the change promoted with the WRS intervention. The same might have occurred to middle management members concerning the organization's benefit of gaining recognition from stakeholders, associated to a sense of pride of working at ISA. Participants' descriptions of feeling used to the WRS intervention and their perception of it as part of the normal work procedures might be a sign of the progress toward an organizational culture that incorporates safety and an attribute of the organizational safety climate, as presented by Zohar (25).

The suggestion that a safety climate was emerging is also relevant for the next main theme, “Culture of care.” Continuous education and follow-up to generate and sustain change were given great importance by all participants. They described that there had been a change in the traditional way of working, focused exclusively on production indicators, toward an increasing consideration of health prevention. The individual process of becoming aware of the importance of care for health, as described by participants, seemed to be perceived as something transferable downwards, from upper positions to the lower organizational levels. “Becoming aware” was sometimes depicted as the need to abide by the rules (“.become aware that they have to.”). It appeared that foremen and probably also cutters had received a two-fold message. The WRS intervention, while addressing through education the individual's lack of health literacy and appealing for personal responsibility to take care of the own and others' health, seemed to be also experienced as an order that has to be obeyed. For manual workers in California, “.the act of drinking water manifests more often as something identified as a requirement of the employer or a requirement of the job, an imposition from a source of power and control rather than an internal impetus” [(10): p. 19]. Although at a different organizational level than the workers in California, some foremen seemed to have experienced the implementation similarly, as a requirement from higher positions that they must adhere. On the other hand, a paternalistic view on the cutters, who were sometimes described as children, appeared to be predominating among participants at different levels. A large responsibility for the shortcomings of the implementation was attributed, thus, to their lack of health literacy and careless health behavior. Accordingly, sanctions were perceived as an enabler to cutters' behavioral change and compliance.

The predominance of a top-down approach with a strong accent in training seemed to be essential for achieving substantial progress in heat stress prevention. However, moving beyond imposition and rational education strategies might be necessary to facilitate behavioral change originated from the internal impetus of individuals. Behavioral change occurs when individuals participate in their own reeducation of beliefs (38, 39). Bennis (40) stated that “.participation is essential for building the partnership, trust, and commitment, which are thought to be vital for long-term performance improvements” [(17): p. 55]. Thus, besides current efforts in pursuing compliance, further developing participation strategies might be beneficial for both foremen and cutters' adherence to the WRS intervention.

Furthermore, most foremen expressed the need to get support in the field, motivation, and enhanced training for themselves and their groups of cutters. These perceptions might affect foremen's perceived control and ability to support the preventive measures. Moreover, perceived control and support in the workplace are well-known factors for well-being at work (41). On the other hand, in Florida, the lack of reward from the employer to manual workers' heat safety behaviors was identified as a primary obstacle to health protection (11). Although cutters were not interviewed at ISA, foremen's need for practical support and motivation might relate to the need for more reward, or incentives, to their health promotion behavior, in addition to the motivation obtained from seeing improved health among their dependents.

In the third main theme, “Traditional production culture vs. Culture of care,” the tension perceived between the traditional way of producing and incorporating health is highlighted. Also, there seemed to have been different priorities in different areas of the organization, and foremen could receive seemingly contradictory instructions. Thus, the enactment of health prevention prioritization appeared sometimes to be inconsistent with policies and guidelines, as described by Zohar (25) in relation to organizational safety climate. Production areas' members might have influenced the staff toward prioritizing the production over the implementation of the WRS preventive measures, which might have affected foremen discern of what behaviors were expected from them, rewarded, and supported. Likewise, in addition to middle management, some foremen were perceived to not be sufficiently involved or be lacking commitment with the WRS intervention. The resulting actions by these individuals might also be perceived by the lower levels as gaps between espoused and enacted priorities, as described by Zohar (25). A previous study has found that when Latino agricultural workers in California categorized employers into those who care for their health and those who do not, it was important for them how a supervisor expressed or acted upon heat safety regulations (10). Moreover, the task overload for foremen and some health promoters at ISA, seemingly associated with the withdrawal of the *boleros* (specialized operators that helped with the implementation in the field), might have given a discordant message to field workers (cutters, health promoters, foremen, and field monitors), as the allocation of resources is considered part of employees' perceptions of top management's safety commitment (29).

On the other hand, a safety climate (25) appeared to have been strengthened by the fact that production levels had not declined following the WRS intervention. This obstacle to cutters' adoption of WRS measures seemed to have been mostly overcome and to have facilitated the implementation of the WRS preventive measures substantially. However, the piecework pay system appeared to be associated with difficulties pursuing compliance among cutters. Mitchell et al. (42) found that Latino farmworkers who were paid piecework in California had a higher physical activity than those who did not and an increased risk of heat-related illness on hot days. As the employer incentivizes higher physical activity through this payment system (10), ISA's manual workers might be put in an ambivalent situation. They are offered immediate monetary incentives to personal production vs. the risk of harm in the future, which is to be subjectively assessed by the workers. Individuals belonging to a population group on the brink of poverty might accordingly opt for the behaviors that will allow them to make their salaries and secure their jobs. Similarly, since hours of work have been reduced with WRS intervention, the duration of exposure to heat might be replaced with a higher work intensity through the piecework system. Cutters might feel they have to work harder to make their piece-rate goals in the fewer hours per day they currently have. Moreover, an indirect effect of the WRS intervention can be that workers who receive the preventive measures feel better, which would allow them to compensate the loss of hours with harder physical work, undoing part of the beneficial effect of the intervention.

Furthermore, as piecework has been identified to influence manual workers' beliefs system in ways that hinder the adoption of self-care practice (10), at ISA, piecework might be affecting training efforts. In the study from California, piecework acted as an incentive for the workers to demonstrate fortitude and be seen as “good workers,” even when suffering from early symptoms of heat illness (10). In a study from Brazil, sugarcane cutters' expectations of getting their salaries could influence their understanding of mental and physical distress symptoms (43). Moreover, this system was found to affect workers' perceptions of their supervisors, who were perceived differently depending on the pay structure. When paid piecework, workers viewed their supervisors as a hindrance in their potential to earn wages because of the interruptions of their work to take breaks and drink water (10). Not unlikely, piecework pay might influence how cutters perceive foremen and health promoters' efforts to encourage and enforce heat stress prevention at ISA.

The fourth main theme, “The importance of the formalization of care,” relates to the organizational infrastructure individuals perceived to favor the WRS implementation, which is a crucial component of IRFOCH (16). Policies, guidelines, procedures, and material and human resources allocated to the WRS intervention appeared to be seen by the study participants as signals of top management's safety commitment and support. This is in line with Bowers et al.'s (29) framework in leadership and safety, as they described employees' perception of top management commitment to health and safety in terms of allocation of resources to safety and inclusion of safety in the structure of the organization. In the current study, the Occupational Health Department was given more weight and power in the organization. Also, top management's active participation and follow-up on the WRS intervention were highlighted by participants, which relates to employees' perception of top management commitment in terms of their active participation in safety (29). Likewise, the perception of a committed top management might have facilitated IRFOCH (16) at ISA.

Lastly, regulations and procedures seemed to be supporting safety climate perceptions at ISA. They appeared to provide individuals with what is expected from them and with the means to do it effectively, which might, as well, relate to their change-specific efficacy beliefs. This seems to confirm the significance of incorporating heat safety in the organizational structure at the mill. Nevertheless, further work appears to be necessary. One participant highlighted the need to institutionalize workers' health outcomes as a measure of individual performance, in the same way that production indicators are used. Measuring and rewarding performance on health outcomes and reduced incidents of adverse events at ISA might reinforce organizational accountability, which is the target that Morera et al. (11) suggested to overcoming structural factors hindering heat safety efforts in the agricultural industry. Also, this might help boost a safety climate substantially, similarly to how area indicators on cutters' compliance with protective equipment use appeared to do at present.

#### Implications

Discretionary differences in low and middle management indicate that more efforts are needed to align production and health protection goals. This and other implementation challenges suggest that to have this health prevention intervention fully internalized and complied with by employees at different levels, employees need to perceive that the company manages and rewards on health metrics besides traditional metrics of efficiency and productivity. A performance assessment process further integrating health metrics might entail the opportunity to align production and health while incorporating institutional incentives to heat stress prevention behavior. Still, to facilitate behavioral change from the internal impetus of individuals, including participation of all organizational levels, in the design and improvement of processes involving health protection, would be suitable, as beliefs can only be reeducated through the involvement of employees (39). These actions might help strengthen organizational accountability, as further responsibility would be given to those with the power and ability to promote and ensure health protection, and more control would be given to those in the lowest organizational hierarchy levels. However, if not addressed, the piecework payment system might weaken organizational accountability and block workers and, subsequently, the management's heat stress prevention efforts. Changing to a stable payment regime, on the other hand, is a difficult transition for any industry in low- and middle-income countries, and concerns around this topic are understandable. However, it may be necessary to explore further options that place workers' health behavior at the center. Finally, the findings indicate the need for enhanced education and support for the foremen, as well as a reduced workload for them and health promoters.

At a national policy level, the study's findings accentuate the need for governments to consider legislation that regulates workers' health protection, and incentives that support companies' initial investing and evaluation of heat stress prevention programs. This way, as long as health and production metrics are improved, the former will be more easily considered into the norms of companies' operations. Likewise, ministries of labor and health should facilitate implementation through employers' and workforce education on the need for such programs.

#### Limitations

The study was confined to one organization in Nicaragua, which limits the transferability of the results. This is accompanied by the inherent difficulty in the generalization in qualitative studies. Nevertheless, a qualitative study provides the opportunity for an in-depth understanding of the participant's experiences of the topic. Moreover, for qualitative studies, it is important to add to the accumulation of results by relating the findings to previous research (44). Neither investigator triangulation nor methodological triangulation techniques (45) have been feasible for the study. However, in order to strengthen inter-rater reliability, the analysis has been refined with one of the authors (KG) throughout the entire process. Furthermore, the authors were aware that there was the risk that the participants might have felt unable to speak freely because of worries that there would be negative consequences. The researchers had therefore gained written confirmation from the mill's highest management assuring there would be no consequences from participating in the interviews, and this information was presented and explained to the participants before the interviews started. The fact that the participants did share negative and critical comments regarding the work situation can be seen as an indication that the participants felt able to discuss both positive and negative experiences and perceptions. In addition, the study results were fed back to ISA and the German Investment Corporation with a report written in Spanish. The findings were found very useful and were overall highly received by the mill. They prompted immediate action, including bi-monthly meetings to systematically address deficits in implementation. In particular, the mill committed to addressing the resources allocated to field implementation, the education of manual workers and supervisors, and the management incentives utilized to ensure workers' health is prioritized not only by the company's leadership but all across the management chain.

Further research focusing on the voices of the manual workers and community-level experiences and interpretations of heat stress prevention (i.e., among workers' families, wives, and children) is necessary. This would complement the current findings and probably contribute profoundly to the understanding of enablers and challenges to heat safety in this and other comparable settings within the agriculture industry and other productive sectors.

#### Conclusions

This study has offered an in-depth view of perceptions of heat safety implementation across different organizational levels at ISA, being the first to approach heat stress prevention in Nicaragua from an organizational psychology perspective.

The theme “A worthwhile struggle” showed that the WRS preventive measures were viewed as part of normality after a difficult start. The implementation was perceived to have resulted in positive consequences for workers' health, their families, and the company. Seeing the intervention's tangible benefits appeared to be associated with positive emotions and motivation to continuing with the preventive measures.

“Culture of care” was a further main theme that highlighted the perception of a change in the company from a traditional production culture toward a sustainable production where the care of health was incorporated. Participants highlighted the importance of education on health promotion. Besides, supervision, control, and “follow-up” were viewed as essential for compliance with the WRS preventive measures. In addition, foremen expressed the need for support, incentives, and training.

A further main theme was “Traditional production culture vs. Culture of care,” which showed the tension perceived to exist in the mill between production and health methods. At the same time, the proven fact that production is not affected adversely by incorporating care of health was an enabling factor. However, the piecework payment regime appeared to still hinder some workers from heat safety practice.

The final main theme, “The importance of the formalization of care,” showed the perception that the systematization of information, the formalization of the monitoring of health indicators, and the regularity and organization of the WRS preventive measures following this monitoring had been facilitators. A high commitment from top management was valued and considered essential for the implementation's establishment, development, and continuity. Moreover, having specific guidelines and regulations on implementing heat stress prevention, with a clear definition of goals, procedures, and authority levels to enforce them, were seen as enablers. Consequently, the need to formalize and institutionalize workers' health outcomes as specific key performance indicators for low and middle management's performance assessments was highlighted and considered critical to tackling current implementation challenges.

Discretionary differences in low and middle management resulting in prioritizing traditional production over health appeared to relate to a fair part of the implementation challenges and indicate that more efforts are needed to ensure health protection. Implementing performance assessments with further focus on health metrics might offer the opportunity to level perceived incentives and value of health and production. Additionally, further education efforts and a process-participation focus might be necessary to enhance involvement.

## Data Availability Statement

The datasets for this manuscript are not publicly available because of ethical concerns relating to informed consent and protection of privacy and confidentiality rights. Requests to access anonymized datasets/codebook should be directed to Kristina Gyllensten, kristina.gyllensten@amm.gu.se.

## Ethics Statement

The studies involving human participants were reviewed and approved by Comité de Ética para Investigaciones Biomédicas (CEIB), Facultad de Ciencias Médicas, Universidad Nacional Autónoma de Nicaragua (UNAN—León), FWA00004523/IRB00003342. The patients/participants provided their written informed consent to participate in this study.

## Author Contributions

JG, KJ, and EA-M identified the need for the study and conceived the project idea. KG, KJ, FP-Z, and IW designed the study. IW coordinated data collection. FP-Z collected and transcribed the data, conducted the analysis, translated the excerpts from the interviews, interpreted the results, and drafted the manuscript. KG and FP-Z discussed and refined the results of the analysis and interpretations. FP-Z, KG, JG, KJ, and EA-M reviewed and edited the manuscript. JG contributed to the translation of excerpts. KG and KJ supervised the project. All authors read and approved the final version of the manuscript.

## Funding

This study was funded by La Isla Network, The Swedish Research Council for Health, Working Life and Welfare, and the University of Gothenburg.

## Conflict of Interest

The authors declare that the research was conducted in the absence of any commercial or financial relationships that could be construed as a potential conflict of interest.

## Publisher's Note

All claims expressed in this article are solely those of the authors and do not necessarily represent those of their affiliated organizations, or those of the publisher, the editors and the reviewers. Any product that may be evaluated in this article, or claim that may be made by its manufacturer, is not guaranteed or endorsed by the publisher.

## Abbreviations

CKDnt: chronic kidney disease of non-traditional origin
IRFOCH: individual readiness for organizational change
ISA: Ingenio San Antonio
LIN: La Isla Network
WRS, water, rest: and shade
IPA: interpretative phenomenological analysis
OHS: occupational health and safety
HR: human resources.
